# Mitochondrial DNA lesions and copy number are strain dependent in endurance‐trained mice

**DOI:** 10.14814/phy2.14605

**Published:** 2020-11-15

**Authors:** Heather L. Vellers, Michael P. Massett, Josh J. Avila, Seung Kyum Kim, Jacqui M. Marzec, Janine H. Santos, J. Timothy Lightfoot, Steven R. Kleeberger

**Affiliations:** ^1^ Department of Kinesiology and Sport Management Texas Tech University Lubbock TX USA; ^2^ Department of Health and Kinesiology Texas A&M University College Station College Station TX USA; ^3^ Division of Research Texas A&M University College Station College Station TX USA; ^4^ Department of Sports Science Seoul National University of Science and Technology Seoul Republic of Korea; ^5^ National Institute of Environmental Health Sciences NIH Research Triangle Park NC USA

**Keywords:** exercise training, interstrain variation, mtDNA copy number, mtDNA lesions

## Abstract

In this pilot work, we selected two inbred strains that respond well to endurance training (ET) (FVB/NJ, and SJL/J strains), and two strains that respond poorly (BALB/cByJ and NZW/LacJ), to determine the effect of a standardized ET treadmill program on mitochondrial and nuclear DNA (nucDNA) integrity, and mitochondrial DNA (mtDNA) copy number. DNA was isolated from plantaris muscles (*n* = 37) and a gene‐specific quantitative PCR‐based assay was used to measure DNA lesions and mtDNA copy number. Mean mtDNA lesions were not different within strains in the sedentary or exercise‐trained states. However, mtDNA lesions were significantly higher in trained low‐responding NZW/LacJ mice (0.24 ± 0.06 mtDNA lesions/10 Kb) compared to high‐responding strains (mtDNA lesions/10 Kb: FVB/NJ = 0.11 ± 0.01, *p* = .049; SJL/J = 0.04 ± 0.02; *p* = .003). ET did not alter mean mtDNA copy numbers for any strain, although both sedentary and trained FVB/NJ mice had significantly higher mtDNA copies (99,890 ± 4,884 mtDNA copies) compared to low‐responding strains (mtDNA copies: BALB/cByJ = 69,744 ± 4,675; NZW/LacJ = 65,687 ± 5,180; *p* < .001). ET did not change nucDNA lesions for any strain, however, SJL/J had the lowest mean nucDNA lesions (3.5 ± 0.14 nucDNA lesions/6.5 Kb) compared to all other strains (nucDNA lesions/6.5 Kb: FVB/NJ = 4.4 ± 0.11; BALB/cByJ = 4.7 ± 0.09; NZW/LacJ = 4.4 ± 0.11; *p* < .0001). Our results demonstrate strain differences in plantaris muscle mtDNA lesions in ET mice and, independent of condition, differences in mean mtDNA copy and nucDNA lesions between strains.

## Graphical abstract

The major findings of this study suggest that an inability to adequately increase exercise capacity to a given dose of endurance training (ET) may be partially explained by genetic underpinnings that inhibit mitochondrial adaptations with ET.

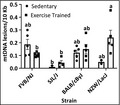

## 1 INTRODUCTION

Cardiorespiratory fitness (CRF) is the most powerful predictor of all‐cause mortality, surpassing typical factors such as smoking, diabetes, and coronary artery disease (Mandsager et al., 2018). CRF is often assumed to be a modifiable risk factor for individuals following physical activity recommendations set by the *National Physical Activity Guidelines for Americans* based on appropriate doses (duration, type, and frequency) of aerobic or endurance exercise (Physical Activity Guidelines Advisory Committee, 2018). Unfortunately, not all individuals increase CRF with a given dose of endurance exercise. Some individuals are highly trainable and increase CRF, while others respond poorly or only marginally (Bouchard et al., 1999). Like the interindividual variation in aerobic capacity trainability in humans, inbred rodent models (e.g., mice and rats; reviewed in Vellers et al., 2018) also demonstrate significant interstrain variation in aerobic capacity trainability. As such, genetic background is a known contributor to the interindividual and ‐strain variation in aerobic capacity trainability with endurance training (Avila et al., 2017; Bouchard et al., 1999).

Because endurance training (ET) adaptations involve complex interactions among multiple body organs and systems, the molecular mechanisms underlying the significant interindividual and ‐strain variation with CRF, or aerobic capacity, trainability is poorly defined. However, at the cellular level, mitochondria are foundational for providing the required energy to cells and tissues to meet the energy demands of exercise (Tarnopolsky et al., 2007). With training, mitochondria undergo a series of changes, including increases in their size and numbers per cell, and increased cristae for increased oxygen diffusion that, together, enhance mitochondrial function for more efficient energy production (Lundby & Jacobs, 2016; Tarnopolsky et al., 2007). Given mitochondrial adaptations are critical for increasing aerobic capacity with ET, highlights the importance of considering mitochondrial adaptations as potential factors underlying the significant interindividual and ‐strain change in exercise capacity with endurance ET.

While a host of methodologies can detect mitochondrial health and function, damage to nucleic acid serves as an indirect indicator of oxidative stress. Physical exercise, including aerobic and resistance training, can generate oxidative stress that, in turn, result in nucleic acid damage (reviewed in 8). Prior studies have investigated the relationship between physical exercise‐induced oxidative stress and nucleic acid DNA damage (Leeuwenburgh & Heinecke, 2001; Packer et al., 2008; Radak et al., 2013), although work has yet to investigate ET‐induced oxidative damage to mitochondrial DNA. Mitochondrial DNA (mtDNA) damage could have significant implications on mitochondrial function that negatively influence aerobic capacity trainability via damaged base pairs that code for mitochondrial oxidative phosphorylation or its replication.

In this current investigation, our primary goal was to characterize DNA damage—nuclear and mitochondrial DNA damage—as well as mtDNA copy number in selected mouse strains presenting differential responses in exercise capacity (determined by work, kg/m; 2). Specifically, we predicted that strains with low changes in exercise capacity phenotypes (i.e., change in exercise work) would accumulate more mtDNA lesions and have less mtDNA content relative to strains that respond well and increase exercise capacity phenotype measures with training. mtDNA copy numbers decrease because of damaged or dysfunctional mitochondria with oxidative stress; therefore, we expected mtDNA copies to decrease after ET in low‐responding strains and increase high‐responding strains.

## 2 METHODS

### 2.1 Mice

The mouse tissue samples (plantaris skeletal muscle) analyzed in this study were taken from previously published work by Avila et al. (2017). The protocols used in the study by Avila et al. (2017) conformed to the standards of humane animal care and were approved by the Texas A&M University Institutional Animal Care and Use Committee (AUPs 2010‐245 and 2013‐0223). The present work for this investigation included a total of 37 six to seven‐week‐old male mice were purchased from Jackson Laboratory (Bar Harbor, ME) and allowed at least 1 week to acclimatize to their new environment. The following strains were randomized into sedentary (SED) or endurance training (ET): FVB/NJ (*n* = 5 SED mice and *n* = 5 ET mice), SJL/J (*n* = 2 SED mice and *n* = 5 ET mice)), BALB/cByJ (*n* = 6 SED mice and *n* = 5 ET mice) and NZW/LacJ (*n* = 4 SED mice and *n* = 5 ET mice)) inbred mice (Jackson Laboratory, Bar Harbor, ME) were selected and used from prior work (Massett et al., 2015; Massett & Berk, 2005) that found differential responses to a standardized treadmill endurance training (ET) program (i.e., trainability). Table 1 summarizes strain characteristics in exercise capacity and body composition measures that represent a portion of previously published work by our group (Avila et al., 2017). All mice were group housed in standard caging and allowed food (Standardized Laboratory Rodent Diet) and water ad libitum and maintained at an ambient temperature of 22°C–24°C on a 12‐hr light:dark schedule.

**TABLE 1:** Body, heart mass and exercise capacity before and after endurance training in four inbred mouse strains

Measurement	FVB/NJ		SJL/J		BALB/cByJ		NZW/LacJ	
	SED	ET	SED	ET	SED	ET	SED	ET
Body and muscle mass	(*n* = 5)	(*n* = 5)	(*n* = 2)	(*n* = 5)	(*n* = 6)	(*n* = 5)	(*n* = 4)	(*n* = 5)
Pretraining BM (g)	26.2 (±0.8)^ab^	27.0 (±0.2)^a^	21.7 (±1.1)^de^	20.5 (±0.4)^e^	23.5 (±0.4)^cd^	24.7 (±0.5)^bc^	26.4 (±1.0)^ab^	27.9 (±1.0)^a^
Posttraining BM (g)	29.9 (±0.8)^ab^*	30.1 (±0.6)^ab^*	25.6 (±0.8)^cd^*	23.8 (±0.3)^d^*	27.4 (±0.6)^bc^*	27.3 (±0.4)^bc^*	31.2 (±1.1)^a^*	31.3 (±0.9)^a^*
Change in BM (g)	3.7 (±0.3)^a^	3.1 (±0.8)^a^	3.9 (±0.4)^a^	3.3 (±0.3)^a^	3.9 (±0.5)^a^	2.6 (±0.5)^a^	4.8 (±0.5)^a^	3.4 (±0.7)^a^
Terminal PM:BM (mg:g)	0.72 (±0.05)^abc^	0.88 (±0.09)^a^	0.63 (±0.08)^abc^	0.61 (±0.04)^bc^	0.82 (±0.06)^ab^	0.79 (±0.03)^abc^	0.55 (±0.05)^c^	0.62 (±0.04)^bc^
Exercise capacity								
Pretraining work (kg/m)	2.63 (±0.14)^a^	2.94 (±0.12)^a^	2.95 (±0.23)^a^	2.97 (±0.09)^a^	2.03 (0.09)^b^	1.90 (±0.11)^b^	0.98 (±0.04)^c^	0.98 (±0.04)^c^
Posttraining work (kg/m)	2.50 (±0.46)^b^	5.01 (±0.19)^a^*	3.42 (±0.19)^b^	5.15 (±0.13)^a^*	2.02 (±0.29)^bc^	1.27 (±0.16)^c^	1.16 (±0.08)^c^	1.02 (±0.16)^c^
Change in work (kg/m)	0.13 (±0.44)^c^	2.07 (±0.26)^ab^	0.47 (±0.04)^bc^	2.18 (±0.21)^a^	0.01 (±0.31)^c^	0.64 (±0.26)^c^	0.18 (±0.05)^c^	0.05 (±0.12)^c^

Note

Values are presented as mean ± *SE*. *n* = 2–6 mice/group. SED, sedentary mice; ET, exercise‐trained mice; Pretraining BM, body mass before training; Change in BM, body mass after training minus before training. Terminal PM:BM, the plantaris muscle mass to body mass ratio after training; Pretraining work, exercise capacity before training; Posttraining work, exercise capacity after training; Change in work, exercise capacity after training minus before training. Values not connected by the same letter were significantly different (*p* < .05). * denotes a significant difference between pre‐ and postmeasures in body mass and exercise capacity within a strain by condition (SED and ET). A portion of these data presented in this table is reproduced from previous work (Avila et al., 2017).

### 2.2 Exercise capacity test protocol

Following a 1‐week acclimation period to housing environment, mice were familiarized with running on a motorized rodent treadmill (Columbus Instruments, Columbus, OH) for 2 days as performed in previous work by Avila et al. (2017), Massett et al. (2009, 2015) and Massett and Berk (2005). Each session was approximately 10 min in duration, and mice were ran at 9 and 10 m per minute (m/min) and up a 10° incline. After familiarization, mice performed two graded exercise performance tests separated by 48 hr (two tests are to ensure repeatability). All assessments started at 9 m/min for 9 min then increased from 10 m/min by 2.5 m/min every 3 min. The starting incline was 0° and raised by 5° every 9 min, with a maximal incline of 15°. Exercise was continued until exhaustion, defined as spending greater than 15 consecutive seconds on the electric grid at the rear of the treadmill. At this point, running time (in min) was recorded, and each mouse was removed from the treadmill and returned to their cage. Following the performance of the two exercise graded test for all mice, a 48‐hr rest period was given, then the standardized endurance treadmill training program was initiated and continued for 4 weeks.

### 2.3 Standardized treadmill training protocol

The standardized treadmill training protocol for mice was performed as described in work by Avila et al. (2017). The exercise groups began the standardized treadmill training protocol following the 48‐hr rest period from the exercise performance test. The treadmill training protocol was performed for 4 weeks, 5 days per week for 60 min per day. The target workload for the training protocol was 65% of the maximal workload achieved during the exercise capacity test. The training speed and duration was increased gradually over the first 2 weeks of training. Treadmill and/or lane assignments were rotated each day for each mouse. The sedentary animals completed a pre‐ and postexercise capacity tests but did not undergo the treadmill training program.

### 2.4 Tissue samples

At least 24 hr after the last exercise performance test, mice were anesthetized by intraperitoneal injection of ketamine (80 mg/kg) – xylazine (5 mg/kg) cocktail and euthanized by exsanguination. Plantaris muscles were excised, and wet weights (in mg) obtained before freezing to calculate tissue to body mass ratios. Body mass was measured before each exercise performance test and prior to terminal surgery.

### 2.5 Mouse DNA lesions (mitochondrial and nuclear) and mitochondrial copy number assays

DNA was extracted from plantaris skeletal muscle of mice (*n* = 37) using the DNeasy Blood & Tissue Kit (Qiagen, Carlsbad, CA) in accordance with the manufacturer's instructions. Isolated DNA was quantified with the Qubit™ dsDNA HS Assay Kit on a Qubit Analyzer (Invitrogen, Life Technologies, Grand Island, NY) in triplicate for accurate quantification. All samples were diluted to 3.0 ng DNA/µl in Tris‐EDTA (TE) buffer (Promega; Madison, WI). We then used a quantitative polymerase chain reaction (QPCR) protocol developed by Santos et al. (2006) and Furda et al. (2012) that used gene‐specific primers to assess for mitochondrial and nuclear DNA lesions, as well as mtDNA copy number (Table 2) as provided in their protocols for mice.

**TABLE 2:** Mouse gene targets and primer pairs for QPCR

6.5‐kb fragment from the β‐Pol gene, accession number, AA79582		Sense
MBFor1	5′‐TAT CTC TCT TCC TCT TCA CTT CTC CCC TGG−3′	
MBEX1B	5′‐CGT GAT GCC GCC GTT GAG GGT CTC CTG−3′	Antisense
10‐kb mitochondria fragment		
2,372	5′‐GCC AGC CTG ACC CAT AGC CAT ATT AT−3′	Sense
13,337	5′‐GAG AGA TTT TAT GGG TGT ATT GCG G−3′	Antisense
117‐bp mitochondria fragment		
13,597	5′‐CCC AGC TAC TAC CAT CAT TCA AGT−3′	Sense
13,688	5′‐GAT GGT TTG GGA GAT TGG TTG ATG−3′	Antisense

Briefly, the GeneAmp XL PCR kit was used to prepare the PCRs: 15 ng of total genomic DNA, 1X buffer, 100 ng/μl final concentration of BSA, 200 μM final concentration of dNTPs, 20 pmol of each primer, 1.3 mM final concentration of magnesium, and nuclease‐free water to a total volume of 45 μL. For the mtDNA assays only (mtDNA lesions and copy number), a restriction enzyme digest (New England BioLabs Inc., Ipswich, MA) was used in the following conditions to linearize the mtDNA to enable effective amplification per each sample (40 µl total for triplicate analysis): 2 hr at 37°C; 3.3 µl nuclease‐free water, 5 µl 1X CutSmart® Buffer, 0.5 µl 10X Bovine Serum Albumin, and 1.25 µl HaeII enzyme. Each PCR was started with a “hot start” for 2 min at 75°C prior to adding the enzyme for the short‐ (SMITO; FailSafe Taq Polymerase; Lucigen, Middleton, WI) and long‐mitochondrial gene fragment (LMITO; LongAmp Taq DNA Polymerase; New England BioLabs Inc., Ipswich, MA), and the nuclear gene (β‐Pol; LongAmp Taq DNA Polymerase; New England BioLabs Inc., Ipswich, MA). The QPCR conditions for each primer are provided in the protocol by Santos et al. (2006) and Furda et al. (2012). Experimental controls included a nondamaged control (3.0 ng/µl), a nondamaged 50% control (1.5 ng/µl), and no DNA (TE buffer). A PCR tube containing 1X TE instead of DNA (“no template” control) and a PCR tube containing 50% DNA amount (DNA diluted 1:1 first) was used to ensure optimization of the PCR cycles. A fluorescence reading was then obtained using the FL600 Microplate Fluorescence (Bio‐Tek; Winooski, VT) and DNA lesions and mitochondrial DNA copy number were calculated as described in the protocol by Santos et al. (2006) and Furda et al. (2012).

### 2.6 Statistical analyses

All data are presented as means ± standard error of the mean (*SEM*) for each group. For each assay, experimental sample data are expressed as relative change compared to nondamaged mouse (C57BL/6J) DNA liver samples. Two‐way ANOVA was used to evaluate DNA lesions (mitochondrial and nuclear) and mitochondrial copy number assays. The Holm–Sidak method was used to determine statistical differences between and within strains (FVB/NJ, SJL/J, BALB/cByJ, and NZW/LacJ) and condition (sedentary or exercise), and *p* < .05 was accepted as statistically significant. A Brown–Forsythe method was employed to test for equal variances among the groups for mtDNA lesions, mtDNA copy number, and nucDNA lesions. For all indices assessed, samples were examined as potential outliers based on the ±2 standard deviations beyond the mean of the group (strain and condition); under these conditions, no samples were eliminated from the analyses. For correlation analyses, all possible pairs between nuclear and mitochondrial DNA lesions, and mtDNA copy number, exercise, and body composition variables were analyzed by Pearson correlation. All statistical analyses were performed using JMP Pro 14.0.0. (SAS Institute Inc., Cary, NC) or Prism 5.0 (GraphPad Software, Inc., La Jolla, CA). The number of mice per group used in this current work was based on the number of available samples (plantaris muscle) from mice in prior work by our group (Avila et al., 2017).

## 3 RESULTS

### 3.1 Mitochondrial mtDNA lesions

**FIGURE 1:**
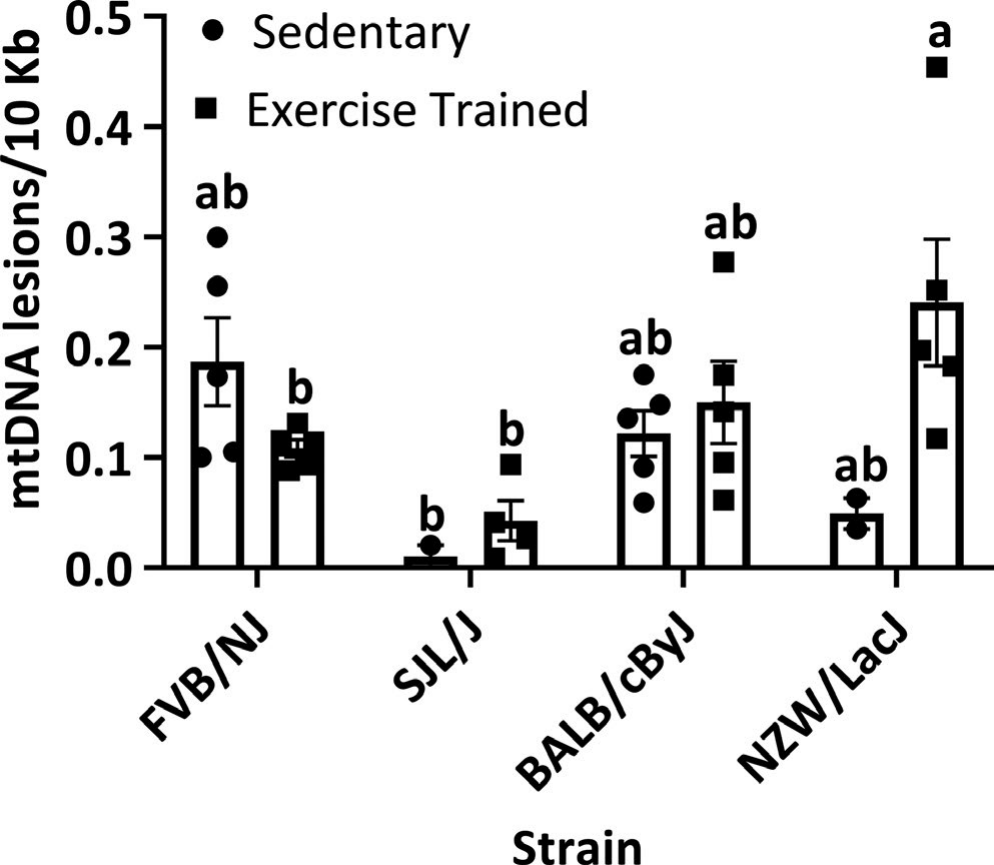
Mitochondrial DNA lesions. SED, sedentary mice (*n* = 5 FVB/NJ mice; *n* = 2 SJL/J mice; *n* = 6 BALB/cByJ mice; *n* = 4 NZW/LacJ mice); ET, endurance‐trained mice (*n* = 5 FVB/NJ mice; *n* = 5 SJL/J mice; *n* = 5 BALB/cByJ mice; *n* = 5 NZW/LacJ mice). There was a significant main effect of strain (*p* = .03), insignificant main effect of condition (*p* = .13), and a significant interaction between the two factors (*p* = .02). Among the SED mice, mean numbers of mtDNA lesions were not significantly different (*p* = .43). In the ET mice, the low‐responding NZW/Lac/J mice accumulated significantly more mtDNA lesions compared to the high‐responding FVB/NJ (*p* = .049) and the high‐responding SJL/J (*p* = .003) strains. Bars not connected by the same letter are significantly different (*p* < .05). Values are presented as mean ± standard error

Among the sedentary mice, mean numbers of mtDNA lesions were not statistically significantly different between the strains (FVB/NJ, 0.19 ± 0.04 mtDNA lesions/10 Kb; SJL/J mice, 0.01 ± 0.01 mtDNA lesions/10 Kb; NZW/LacJ, 0.05 ± 0.01 mtDNA lesions/10 Kb; BALB/cByJ, 0.12 ± 0.02 mtDNA lesions/10 Kb; *p* = .43; Figure 1).

Conversely, in the exercise‐trained mice, we found the low‐responding NZW/LacJ mice (0.24 ± 0.06 mtDNA lesions) accumulated more mtDNA lesions compared to strains that significantly increase their exercise capacity with training: FVB/NJ (0.11 ± 0.01 mtDNA lesions; *p* = .049) and SJL/J (0.04 ± 0.02 mtDNA lesions; *p* = .003) mice (Figure 1).

### 3.2 Mitochondrial copy number

**FIGURE 2:**
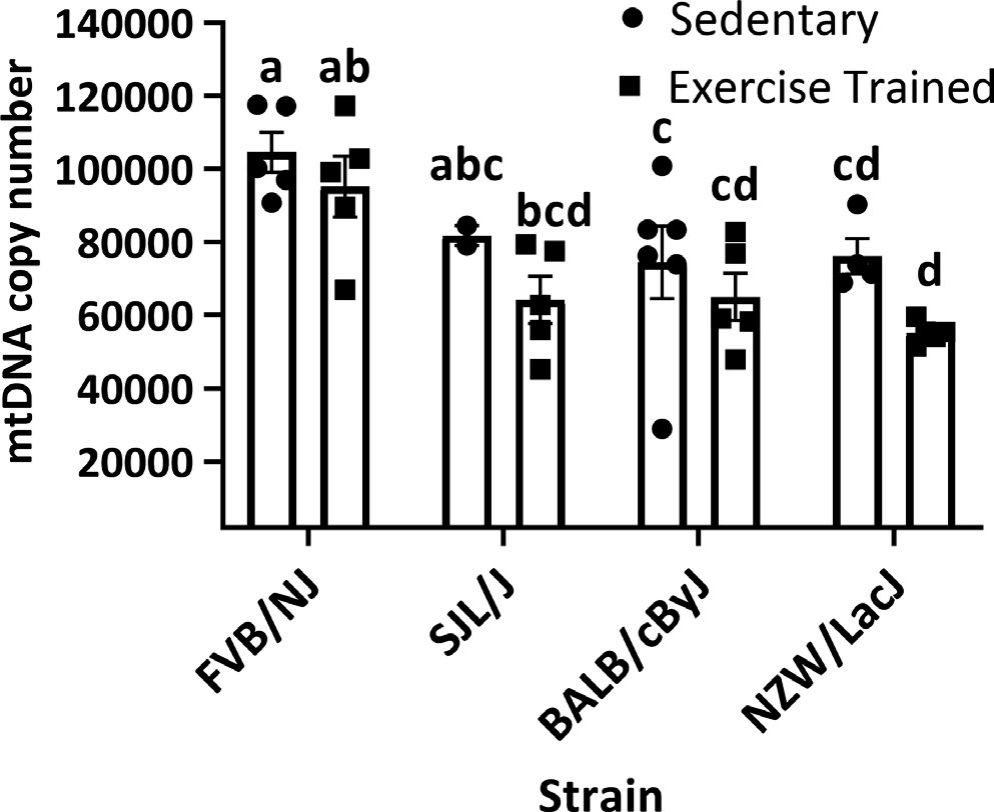
Mitochondrial DNA copy number. SED, sedentary mice (*n* = 5 FVB/NJ mice; *n* = 2 SJL/J mice; *n* = 6 BALB/cByJ mice; *n* = 4 NZW/LacJ mice); ET, endurance‐trained mice (*n* = 5 FVB/NJ mice; *n* = 5 SJL/J mice; *n* = 5 BALB/cByJ mice; *n* = 5 NZW/LacJ mice). The high‐responding FVB/NJ mice (*n* = 5 SED mice; *n* = 5 ET mice) had significantly higher mtDNA copy numbers compared to the other three strains (*p* < .001). The ET mice had significantly less overall mean mtDNA copies compared to SED mice (*p* = .012). The mean mtDNA copies were not significantly different between strains in SED (*p* > .05) or ET (*p* > .05) mice. There was not a significant interaction between strain and condition (*p* = .81). Bars not connected by the same letter are significantly different (*p* < .05). Values are presented as mean ± standard error

In the sedentary mice, we found significantly greater mean mtDNA copy numbers in the FVB/NJ strain (104,564 ± 5,448 mtDNA copies) compared to BALB/cByJ (74,474 ± 9,889 mtDNA copies) and NZW/LacJ (76,155 ± 4,847 mtDNA copies) strains (*p* < .001; Figure 2). Following endurance training, we similarly found mtDNA copy number was significantly greater mtDNA copies in FVB/NJ mice (95,216 ± 8,334 mtDNA copies) compared to the low‐responding BALB/cByJ (65,016 ± 6,442) and NZW/LacJ strains (55,219 ± 1,313 mtDNA copies) (*p* < .001; Figure 2). We did not indentify an interaction between strain and condition in mtDNA copy number (*p* = .81; Figure 2).

### 3.3 Nuclear DNA lesions

**FIGURE 3:**
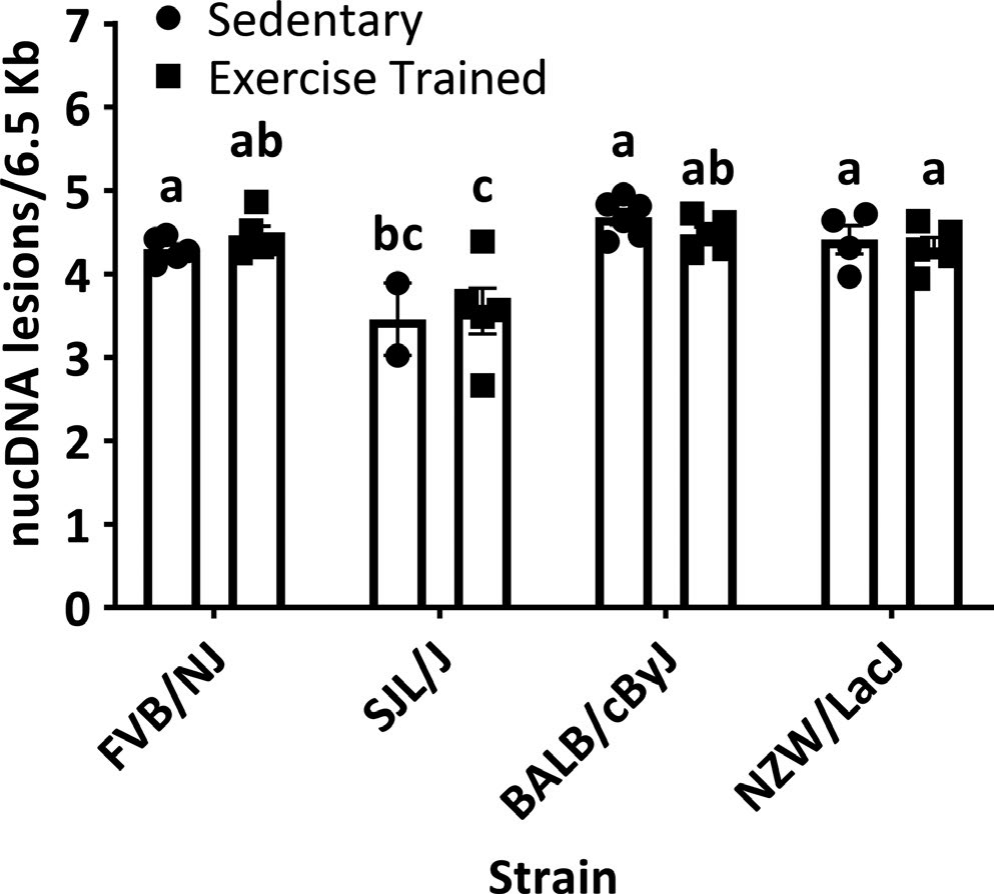
Nuclear DNA lesions. SED, sedentary mice (*n* = 5 FVB/NJ mice; *n* = 2 SJL/J mice; *n* = 6 BALB/cByJ mice; *n* = 4 NZW/LacJ mice); ET, endurance‐trained mice (*n* = 5 FVB/NJ mice; *n* = 5 SJL/J mice; *n* = 5 BALB/cByJ mice; *n* = 5 NZW/LacJ mice). There was a significant difference in nuclear DNA lesions due to strain with the lowest overall lesions found in the high‐responding SJL/J mice compared to all other strains (*p* < .0001). In the SED mice, mean numbers of nucDNA lesions in SJL/J mice were significantly smaller compared to all other strains (*p* < .0001). The mean numbers of nucDNA lesions were not significantly different between the SED and ET strains (*p* > .05), and there was not a significant interaction between strain and condition (*p* = .84). Bars not connected by the same letter are significantly different (*p* < .05). Values are presented as mean ± standard error

Among the sedentary mice, the SJL/J mice had significantly lower mean numbers of nucDNA lesions (3.5 ± 0.14 nucDNA lesions/6.5 Kb) compared to all other strains (FVB/NJ = 4.3 ± 0.11 nucDNA lesions/6.5 Kb, BALB/cByJ = 4.7 ± 0.09 nucDNA lesions/6.5 Kb, NZW/LacJ = 4.4 ± 0.11 nucDNA lesions/6.5 Kb, *p* < .0001; Figure 3). We did not find a significant interaction between strain and condition on nucDNA lesions (*p* = .84; Figure 3). Compared to respective sedentary mice, we found no significant effects of exercise on nucDNA lesions in any of the four strains (FVB/NJ, 4.5 ± 0.11 nucDNA lesions/6.5 Kb; SJL/J, 3.6 ± 0.27 nucDNA lesions/6.5 Kb; BALB/cByJ, 4.7 ± 0.09 nucDNA lesions/6.5 Kb; NZW/LacJ, 4.3 ± 0.12 nucDNA lesions/6.5 Kb; *p* > .05; Figure 3).

### 3.4 Correlations for mitochondrial and nuclear DNA lesions and mitochondrial copy number with exercise capacity and body composition phenotypes

Phenotypic correlations for sedentary mice are shown in Table 3, and correlations for exercise‐trained mice are shown in Table 4. In sedentary mice, we found a significant inverse correlation for mtDNA copy number with change in exercise capacity (change in work, kg/m, *r* = −.49, *p* = .046), and a significant inverse correlation for nucDNA lesions with posttraining work (*r* = −.50, *p* = .04). In exercise‐trained mice, we found mtDNA lesions were inversely correlated with pre‐ (*r* = −.63, *p* = .004), post‐ (*r* = −.58, *p* = .009), and change in exercise work (*r* = −.47, *p* = .04).

**TABLE 3:** Correlations for DNA lesions and mtDNA copy number with exercise capacity and body composition phenotypes in four sedentary mouse strains

	PM:BM	mtDNA lesions/10 Kb	mtDNA copy number	nucDNA lesions/6.5 Kb
Pretraining body mass	−0.03	0.36	0.22	−0.01
Posttraining body mass	−0.10	0.11	0.13	0.09
Change in body mass	−0.20	−0.56	−0.14	0.27
Pretraining work	0.43	0.43	0.40	−0.44
Posttraining work	0.15	0.17	−0.04	**−0.50**
Change in work	−0.25	−0.23	**−0.49**	−0.22
PM:BM		0.54	0.18	0.29
mtDNA lesions/10 Kb	0.54		0.48	0.12
mtDNA copy number	0.18	0.48		−0.09
nucDNA lesions/6.5 Kb	0.29	0.12	−0.09	

Note

Numbers in bold indicate significant correlations (*p* < .05). Change, Difference between posttraining and pretraining values; PM:BM, plantaris mass to body mass ratio. Pairwise correlations were performed using individual sedentary mice consisting of FVB/NJ, SJL/J, BALB/cByJ, and NZW/LacJ strains.

**TABLE 4:** Correlations for DNA lesions and mtDNA copy number with exercise capacity and body composition phenotypes in four exercise‐trained mouse strains

	PM:BM	mtDNA lesions/10 Kb	mtDNA copy number	nucDNA lesions/6.5 Kb
Pretraining body mass	0.25	**0.71**	0.17	**0.62**
Posttraining body mass	0.11	**0.60**	0.10	**0.58**
Change in body mass	−0.33	−0.24	−0.17	−0.10
Pretraining work	0.29	**−0.63**	**0.50**	−0.31
Posttraining work	0.10	**−0.61**	**0.50**	−0.37
Change in work	−0.04	**−0.52**	0.43	−0.37
PM:BM		−0.01	**0.54**	0.30
mtDNA lesions/10 Kb	−0.01		−0.13	0.47
mtDNA copy number	0.54	−0.13		0.40
nucDNA lesions/6.5 Kb	0.30	0.47	0.40	

Note

Numbers in bold indicate significant correlations (*p* < .05). Change, Difference between posttraining and pretraining values; PM:BM, plantaris mass to body mass ratio. Pairwise correlations were performed using individual exercise‐trained mice consisting of FVB/NJ, SJL/J, BALB/cByJ, and NZW/LacJ strains.

We also identified mtDNA lesions were positively correlated with the average pre‐ (*r* = .66, *p* = .002) and postbody mass (*r* = .55, *p* = .02). mtDNA copy number positively correlated with the plantaris muscle weight to body weight ratio (*r* = .54, *p* = .01), and nucDNA lesions positively correlated with the average pre‐ (*r* = .62, *p* = .003) and postbody mass (*r* = .58, *p* = .008). Collectively, these results suggest that greater mtDNA lesions negatively correlate with inherent exercise capacity (work, kg/m) and change in exercise capacity with training.

## 4 DISCUSSION

Prior work has identified a significant contribution of genetic background (up to 50% heritability) in the regulation of aerobic capacity trainability with endurance training in human (Bouchard et al., 1999) and animal models (Avila et al., 2017; Massett & Berk, 2005). The major findings in the present investigation support an underlying mechanism(s) involved in mitochondrial function and DNA repair potentially linked to the interstrain variation in aerobic capacity trainability following completion of a standardized endurance training program. That is, we found greater mtDNA damage in plantaris muscles of mice that do not significantly increase their exercise (aerobic) capacity with training compared to strains that increase their aerobic capacity. This present work is the first to provide preliminary evidence that there are strain‐dependent differences in mtDNA lesions with ET, and differences in mtDNA copy number and nucDNA lesions across strains independent of exercise training.

In plantaris muscle samples from sedentary mice, we did not find statistically significant differences in the plantaris muscle mtDNA lesions between the strains (Figure 1) or correlations with measures of exercise capacity (Table 3). However, from muscle samples in the trained state, we found an inverse correlation between mtDNA lesions and exercise capacity (Table 4), suggesting that endurance training decreases mtDNA lesions. Among the strains, we found significantly higher mtDNA lesions in the low‐responding NZW/LacJ mice compared to strains that did increase their exercise capacity with ET (FVB/NJ and SJL/J mice). Although, in the other low‐responding strain—BALB/cByJ—we found similar mean mtDNA lesions as the high‐responding FVB/NJ and SJL/J ET mice. It is unclear why one low‐responding strain had higher mtDNA lesions compared to high‐responding strains, and one did not, although it must be considered that there are various interstrain differences in phenotypes. In this current work, we considered DNA lesions and mtDNA copy number in the plantaris muscle. Given that within a strain, DNA lesions and mtDNA copy number can vary across tissues, leave open the possibility for other affected tissues not considered in this current work. Thus, our current findings should be interpreted as unique strain‐dependent characteristics in sedentary and ET mice, and with the understanding that further work is needed to analyze these features across other tissues and strains.

Complementing our findings with the effect of endurance training on mtDNA lesions, we found an inverse correlation of mtDNA copy number and change in exercise capacity with training (Table 4). While it is beyond the scope of our findings to speculate reasons why endurance ET would decrease mtDNA copy number, there are several possibilities for this relationship including cellular processes, such as mitophagy, that are either still adapting to the stress response of exercise and eliminating mtDNA through selective mitochondria degradation, or experiencing an overabundance of exercise‐induced oxidative stress thereby reducing mtDNA copies alongside damaged and dysfunctional mitochondria. However, the fact we observed an inverse correlation with the change in exercise capacity and mtDNA copy number suggests a healthy homeostatic balance between endurance ET‐induced oxidative stress response. Thus, we suspect that increasing the length of training (e.g., 20 weeks) would eventually lead to increased mitochondrial biogenesis, and hence, mtDNA copy number.

Among the strains, we found significantly higher overall mean mtDNA copy numbers in the FVB/NJ mice—a strain with significant increases in exercise capacity with ET—compared to all others, independent of ET (Figure 2). Few studies that have investigated the link between mtDNA copy number and exercise in humans (Baykara et al., 2016; Chang et al., 2016) and in rodents (Cao et al., 2012) have demonstrated that mtDNA copy numbers are higher in those that are highly trainable. For example, in skeletal muscle of mice (gastrocnemius), Cao et al. (Cao et al., 2012) found that a swimming endurance regimen (5 days/week; 20 weeks) significantly increased copy numbers with 30 min of exercise per day. Compared to our current study methods and design, it is important to note that we prescribed a 4‐week standardized endurance training program, while the work by Cao et al. (2012) implemented a 20‐week training regimen. Thus, because the mtDNA copy number changed following 4 weeks of ET, it is possible that within and between strain changes with ET would be greater if we increased the length of the training protocol. Furthermore, the fact that we analyzed a predominantly fast‐twitch muscle (plantaris muscle) DNA lesions and mtDNA copy number, compared to the gastrocnemius (mixed fiber type distribution) analyzed by Cao et al. (Cao et al., 2012), could also explain why our study did not find increases in mtDNA copy number with training. In humans, Baykara et al. (2016) found that mtDNA copy number was significantly higher in highly trained swimmers compared to normal and untrained swimmers in peripheral blood lymphocytes. Another study in humans by (Danese et al., 2017) found in postmenopausal women that regular daily aerobic exercise (at least 150 min per week for at least 6 months) associated with higher mtDNA copy numbers in leukocytes. When comparing the effect of ET on skeletal muscle mtDNA copy number, Pino et al. (2019) found that while ET increased mitochondrial respiration in individuals with type 2 diabetes, it was not accompanied by increases in mtDNA copy number, This finding (Pino et al., 2019) is in agreement with our results in that ET did not change mtDNA copy. Caution must be taken in relating our findings gathered from skeletal muscle to the human work by Baykara et al. (2016) and Danese et al. (2017) because it is unclear whether exercise‐induced changes in mtDNA copy number are differentially regulated across tissues and/or cell types. Taken together, the increased mtDNA lesions we found, coupled with lower overall mtDNA copy number in response to ET, suggests mitochondria in low‐responding strains may be predisposed to generate detrimental levels of mitochondrial‐derived oxidative damage with exercise, a potential factor inhibiting the ability to increase exercise capacity with training.

We also investigated strain‐ and exercise‐dependent effects on nucDNA lesions. Across the sedentary (Table 3) and exercise‐trained (Table 4) mice, we found an inverse correlation between nucDNA lesions and with posttraining exercise capacity. In considering strain comparisons, the SJL/J strain had the lowest overall average nucDNA lesions compared to all others, and this was irrespective of ET (Figure 3). Importantly, because we found higher mean numbers of nucDNA lesions in the other strain that similarly increased exercise capacity with ET (FVB/NJ strain), and because ET did not change nucDNA lesions, we suggest nucDNA lesions are likely not a result or inhibitor for increasing exercise capacity with training. Because DNA lesions are tissue‐ and cell specific, it is possible that other tissues and organs may have changes in nucDNA lesions with ET. For example, Radák et al. (2002) found that ET decreased nucDNA damage in the gastrocnemius muscle of aged rats. One likely explanation for the different effects of ET on nucDNA lesions is because the plantaris muscle is predominantly composed of fast twitch (type 2 muscle fibers) and the gastrocnemius, a mixed muscle fiber type distribution (type 1 and type 2). In human lymphocyte samples taken, following the most strenuous endurance training event—the Ironman triathlon—Reichhold et al. (2008) found no acute nucDNA damage in Ironman athletes. Thus, like mtDNA damage and copy number assessments, the sample type plays an integral role in changes in nucDNA lesions after ET.

A limitation of our study is the small number of strains incorporated into our analyses. Future work inclusive of more inbred mouse strains (e.g., ~24 used in the study by Avila et al. 2) and mice per strain is needed to increase power to determine the effect, and interactions, of strain and ET with DNA lesions and mtDNA copy number. While the inclusion of more strains and mice within each strain is necessary to assess the reproducibility of our current findings, our results provide a foundation for future work involving the interplay between genetic background and its regulation on mitochondrial function and DNA repair mechanisms with ET adaptions. Because our findings indicate strain differences in critical aspects of the mitochondrial genome prompt further work to investigate other aspects such as its major DNA sequence. It is now known that genetic contribution to phenotypes is not only driven by the nuclear genome but an interplay between the nuclear and mitochondrial genomes (Baris et al., 2017). Thus, the differences in mtDNA lesions and copy number may not only be influenced by associated nuclear genes but also mitochondrial genes that may associate and interact with nuclear mitochondrial‐encoded genes to regulate a phenotype such as response to ET. Another potential study limitation is the sample we utilized to assess DNA lesions and mtDNA copy number. Skeletal muscles vary according to fiber type, and it is unknown whether the fiber composition within or between the strains analyzed in this current study affected our results. However, the procedure used to isolate DNA from the plantaris muscles were taken from the whole muscle tissue and were homogenized appropriately. Finally, this current investigation only included males. Thus, future work should include females to determine whether sex differences exist among strains following ET.

In summary, this work suggests that an inability to increase aerobic capacity to a given dose of ET adequately may be, at least partially, explained by genetic underpinnings that inhibit mitochondrial adaptations with training. Thus, based on our current data suggesting strain‐dependent mtDNA lesions and copy numbers accumulated with and without ET, there may be other aspects of the mitochondrial genome that work together to regulate the ability to respond to ET. Future investigations should incorporate makers of mitochondrial‐derived oxidative stress (e.g., plasma and/or skeletal muscle cell‐free mtDNA), mitophagy (PARKIN and PINK1 protein expression), and mitochondrial biogenesis (e.g., PGC‐1α gene expression) and fission (Drp1 protein expression) and fusion (Mfn1, Mfn2, and Opa1) to determine ET‐induced changes to mitochondria across a diverse panel of inbred strains.

## CONFLICT OF INTEREST

No conflict of interest, financial or otherwise, are declared by the authors.

## AUTHOR CONTRIBUTIONS

J.J.A. and S.K.K. performed the exercise capacity tests, endurance training regimen, and tissue collection in all mice. M.P.M provided plantaris skeletal muscles of mice that were utilized in previously published work (Avila et al., 2017). With these samples, H.L.V conceived and designed the research presented in this current work with oversight from S.R.K. and J.T.L., interpreted results of experiments, analyzed data, prepared figures, and drafted the manuscript. J.H.S. developed the DNA lesions (mitochondrial and nuclear) and mitochondrial copy number and assisted with the interpretation of the results. J.M. helped optimize the assays and interpretation of the results. All authors edited and approved the final version of the manuscript.
